# Death of Rasori

**Published:** 1837-10-01

**Authors:** 


### 2. DEATH OF RASORI.

Born in 1766, Rasori died on the 15th of April, 1837, at the age of 71 years.

His life was a checquered one, and he suffered in the political troubles of his injured and unhappy country.

He was born at Parma, where his father was head apothecary to the principal hospital. At the age of eight he entered the university, in which he acquired much general knowledge. At the age of 19 he was made doctor of medicine by the University of Pisa, by which he was sent abroad to study surgery. He departed for Florence, where he became the friend and pupil of Fontani, Nannoni, and Mascagni. In three years he removed to Pavia, where he remained two years, and enjoyed the friendship of Spallanzani, whom he assisted in his experiments.

At this period the Brunonian doctrines made their appearance in Italy. Rasori studied Brown's work with ardour, and translated it into his own language, arguing at the same time against many of the doctrines which it contained. In 1793, Rasori came to England, where he remained for two years, and on his return settled at Milan.

In 1796, Rasori was appointed professor in the University of Pavia, in the room of Scarpa, who refused to take the oath of allegiance to the government of Bonaparte.

After having been professor for two years, he was appointed secretary to the Minister of the Interior. Not liking, however, this situation, he returned to Pavia in the capacity of professor of clinical medicine, and chief of the medical directory. He was deprived of this office in three months, and replaced by his rival Moscati.

He was now made government superintendent of the great hospital of Milan, but immediately retired with the French army to Gênes, where he remained until its surrender. During the siege a petechial fever raged generally and extensively, and Rasori treated it according to the principles which he had already explained in his clinical lectures at Pavia. He was very successful, and published a history of the disease, in which he gave an account of the doctrine of contra-stimulants, and the employment of large doses of tartar emetic. After the battle of Marengo he returned to Milan.

In 1807, Rasori's reputation having become established, strangers began to resort to him, and he was allowed by the government to form two clinical wards, one for men, and the other for women, each of more than 100 beds, in the great hospital of Milan. It was in these wards that he practically developed his system, and instructed the pupils, who afterwards spread his doctrines so widely.

He was soon appointed first physician of the kingdom. The petechial fever was then raging. Rasori stopped its ravages by isolating the affected.

In 1808, the government made him professor of clinical medicine to the military hospital for the instruction of medical officers for the army. To oppose the representations of the enemies of his doctrine, Rasori published annual reports of the results of his practice.

On the re-occupation of Lombardy by the Austrians, Rasori lost all his appointments, and fell under the suspicion of government. On a conspiracy being apprehended, he was seized, imprisoned, and kept four years in confinement. During his imprisonment, although his health suffered considerably from intermittent fever, he displayed his wonted activity of mind; he wrote a review of a work on diseases of the spine, translated a work from the German, and began to prepare a large work on inflammation, which he lived to complete before his death. This work is about to be published, and has excited a good deal of expectation.

After his release, his hospital practice was as much followed by pupils as before, and his private practice was large. Among others, the late Princess of Wales, when in Italy, summoned him during illness to her assistance.

A colossal statue of him in marble is about to be erected in Milan.

Rasori's doctrines have been too often and too deeply canvassed by us, to require any notice at our hands at present. They will probably sleep with their author.

---

We regret to announce the death of Dr. D. Uwins.

